# Hepatitis B virus reactivation in seronegative occult hepatitis B patient receiving ibrutinib therapy

**DOI:** 10.1186/s12985-023-02140-w

**Published:** 2023-08-01

**Authors:** Lok-Ka Lam, Thomas Sau Yan Chan, Yu-Yan Hwang, Lung-Yi Mak, Wai-Kay Seto, Yok-Lam Kwong, Man-Fung Yuen

**Affiliations:** 1 Department of Medicine, School of Clinical Medicine, The University of Hong Kong, Pokfulam, Hongkong; 2 State Key Laboratory of Liver Research, The University of Hong Kong, Pokfulam, Hongkong; 3 Queen Mary Hospital, Professorial Block, 102, Pokfulam Road, Hong Kong

**Keywords:** HBV reactivation, Ibrutinib, B cell depletion, Occult hepatitis B, Case report

## Abstract

**Background:**

Ibrutinib is a Bruton’s tyrosine kinase (BTK) inhibitor approved for the treatment for several mature B-cell malignancies. Reactivation of hepatitis B virus (HBV) is a well-described complication in patients with chronic HBV infection or prior HBV exposure undergoing cytotoxic or immunosuppressive chemotherapy for hematologic malignancies. This phenomenon has been frequently reported with rituximab. However, published data on the risk of HBV reactivation induced by ibrutinib are scarce. Cases of HBV reactivation in hematologic patients receiving ibrutinib therapy have recently been described, but limited only to overt hepatitis B patients or seropositive occult hepatitis B patients.

**Case presentation:**

We report the first case of HBV reactivation during ibrutinib treatment in an asymptomatic 82-year-old woman with seronegative occult hepatitis B patient (i.e., negative for HBsAg, anti-HBc and anti-HBs). Four months after ibrutinib treatment, her liver function test (LFT) was deranged, with seroconversion to HBsAg positivity. Serum hepatitis B virus DNA was quantified to be 1.92 × 10^8^ IU/ml. Antiviral treatment was initiated, and viral load was gradually suppressed with improvement in LFT.

**Conclusions:**

Our case illustrated that in populations with a high incidence of HBV exposure, systematic screening for HBV exposure is essential prior to ibrutinib treatment, followed by serial monitoring of serologic and molecular markers of hepatitis B. There is a need for an international consensus to support the recommendation of antiviral prophylaxis against HBV reactivation in patients using ibrutinib.

## Background

Reactivation of hepatitis B virus (HBV) is a well-described complication in patients with chronic HBV infection receiving immunosuppressants. Currently, patients with chronic HBV infection (positive for hepatitis B surface antigen, HBsAg+) or past exposure to HBV (positive for antibody to hepatitis B core antigen, anti-HBc+) should be risk-stratified based on the type of immunosuppression. The risk of reactivation can be broadly classified into high risk (>10%), moderate risk (1–10%), and low risk (< 1%) [1, 2]. HBsAg + or HBsAg-/anti-HBc + subjects are at high-risk (> 10%) of HBV reactivation if they receive B-cell depleting therapy or undergo hematopoietic stem cell transplantation. Ibrutinib is a Bruton tyrosine kinase (BTK) inhibitor approved for the treatment of various mature B-cell malignancies [3]. Cases of HBV reactivation in hematologic patients receiving ibrutinib therapy have been recently described, both in HBsAg + and HBsAg-/anti-HBc + patients [4]. Here we report the first case of HBV reactivation in a seronegative occult hepatitis B patient receiving ibrutinib.

## Case presentation

A 61-year-old woman presented in November 2000 with chronic lymphocytic leukaemia (CLL). She was treated with fludarabine, mitoxantrone and dexamethasone, followed by chlorambucil from February 2001 to June 2002.

She remained well until 2021, when she presented with a tonsillar mass that on biopsy showed small lymphocytic lymphoma (SLL). She received the anti-CD20 antibody rituximab, but with the first infusion could only tolerate less than 10% of the intended dose before a serious infusion reaction aborted the treatment. The treatment was then changed to ibrutinib (420 mg/day). Prior to initiation of rituximab, she was negative for HBsAg and anti-HBc. Serum HBV-DNA was not tested.

### Investigations / diagnosis / treatment

Four months after ibrutinib treatment, her alanine aminotransferase (ALT) level suddenly increased to 350 U/L. HBsAg became positive, with HBV DNA quantified at 1.92 × 10^8^ IU/ml, confirming HBV-related hepatitis. She had not received any blood product in the prior months, and there was no known HBsAg + household contacts. Antibodies against hepatitis A, hepatitis C and hepatitis E viruses were negative. Ultrasonography did not show any liver pathology. With no prior clinical or serological evidence of HBV exposure in recent years, a diagnosis of HBV reactivation from seronegative occult hepatitis B infection was made. Entecavir 0.5 mg/day was commenced.

### Outcome and follow up

**Fig. 1:**
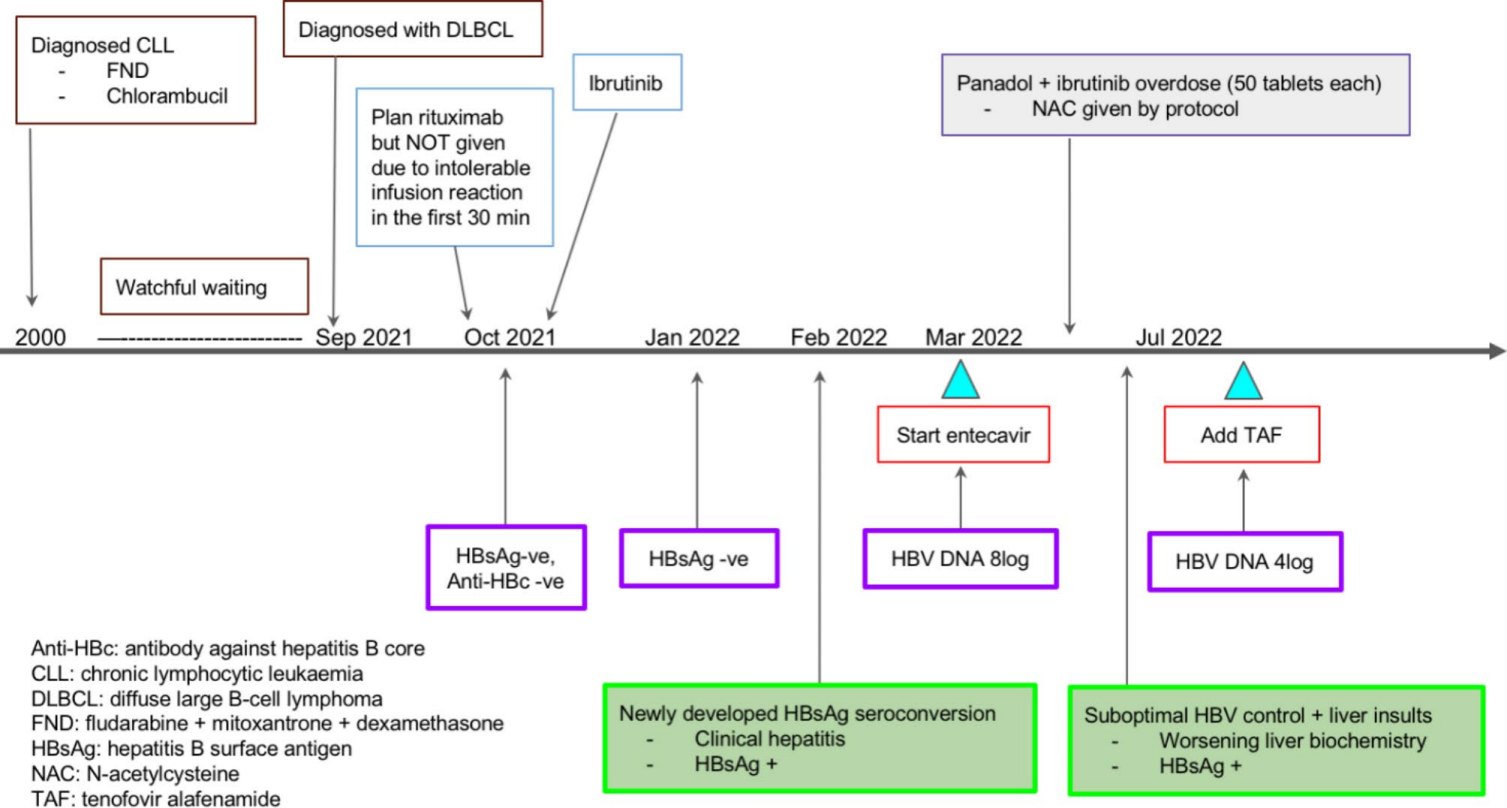
Summarized course of the haematological disease and HBV reactivation

With entecavir treatment, her liver function improved and HBV DNA level declined from 1.92 × 10^8^ IU/ml to 33,900 IU/ml over 4 months. The clinical course was complicated by an intentional overdose of paracetamol (exact dose ingested unknown). ALT rose to 1956 U/L, and aspartate aminotransferase increased to 1324 U/L. There was improvement with N-acetylcysteine. However, her serum HBV DNA was still high at 33,900 IU/ml. Tenofovir alafenamide 25 mg/day was added for better HBV control. At the latest follow-up 6 months after the initial episode of HBV reactivation, she had remained asymptomatic, with ALT at 180 U/L and HBV DNA at 2450 IU/ml (Fig. 1). Her blood values obtained before, during, and after HBV reactivation were shown in Table 1.

**Table 1:** Blood values obtained before, during, and after reactivation

			6/5/2008 (at age 70)	14/10/2021 (before ibrutinib)	15/2/2022 (HBV reactivation)	15/3/2022	6/5/2022	27/7/2022 (paracetamol and ibrutinib overdose)	30/8/2022	13//9/2022
Bilirubin (umol/L)			7	7	**12**	6	8	30	10	9
ALP (U/L)			48	54	**251**	61	57	52	81	71
ALT (U/L)			18	12	**350**	79	141	1956	180	29
AST (U/L)			17	16	**202**	61	89	1324	203	29
GGT (U/L)			N/A	N/A	N/A	N/A	N/A	58	57	47
HBVDNA			N/A	N/A	N/A	**1.92 × 10^8**	**6.29 × 10^5**	**3.39 × 10^4**	**2.45 × 10^3**	N/A
HBsAg			N/A	**-ve**	**+ve**	N/A	N/A	+ve	N/A	N/A
Anti-HBc			N/A	**-ve**	N/A	N/A	N/A	N/A	N/A	N/A
Anti-HBe			N/A	N/A	N/A	N/A	N/A	-ve	N/A	N/A
						Added entecavir		Added tenofovir		

Abbreviations: ALP: Alkaline phosphatase ; ALT: Alanine aminotransferase ; AST: Aspartate aminotransferase ; GGT: Gamma-glutamyl transferase; HBsAg: Hepatitis B surface antigen; HBVDNA: Hepatitis B virus DNA level; anti-HBc: Hepatitis B virus core antibody; anti-HBe: Antibody to hepatitis B e-antigen; N/A: not available.

## Discussion and conclusions

The incidence and severity of HBV reactivation is related to the degree of immunosuppression. Pre-emptive antiviral prophylaxis has been shown to be effective in preventing virologic relapse. Guidelines generally recommend that patient receiving immunosuppressive or cytotoxic therapy should be screened for HBsAg, anti-HBc, and/or HBV DNA [5, 6]. Treatment can be either prophylactic or pre-emptive. Patients with positive serum HBsAg should be treated with antiviral prophylaxis for up to 12–18 months after completion of immunosuppressive therapy [6]. HBV reactivation in patients with prior HBV exposure (HBsAg-/ anti-HBc+), however, remains a debatable issue. It is suggested that patients receiving high-risk immunosuppressive regimens are treated similarly to patients with chronic HBV.

Ibrutinib is a relatively new agent targeting BTK in the B-cell signalling pathway. The summary of product characteristics of ibrutinib states that HBV reactivation may occur during treatment and recommends serological testing for HBV and hepatitis C virus prior to treatment [7]. The national Veterans Health Administration [8] identified seven cases of HBV reactivations among 444 at-risk patients, which corresponds to a cumulative incidence of 1.5% in patients with positive anti-HBc irrespective of HBsAg or HBV DNA status; indicating a moderate risk of HBV reactivation [9, 10]. Our case report is consistent with existing literature regarding the risk of HBV reactivation in ibrutinib-treated subjects who were previously HBV-exposed.

It is noteworthy that our patient’s hepatitis serology was both anti-HBc and HBsAg negative (although HBV DNA was not checked) prior to initiation of ibrutinib. As IgM anti-HBs antibodies were not measured, acute hepatitis B may not completely be excluded in the present case. Nonetheless, based on detailed clinical history, the fact that she developed HBV reactivation suggests that she was most likely a seronegative occult HBV patient. Anti-HBc antibodies might have waned over time spontaneously and further precipitated by the prior use of fludarabine, which is a well-known lymphodepleting agent [11]. It has been well-recognised that seronegative (i.e., negative for anti-HBc and antibody to HBsAg) occult HBV infection accounts for up to 20% of all occult HBV cases [12, 13] .

Our case demonstrates that systematic HBV screening (including HBV DNA) is essential before starting treatment with ibrutinib. In populations with a high incidence of exposure to HBV, it is of paramount importance to monitor serological and molecular markers of hepatitis B in patients treated by ibrutinib. Moreover, there is a need for more data to confirm the risk of HBV reactivation upon ibrutinib treatment, thereby arriving at an international consensus to support the recommendation of antiviral prophylaxis against HBV reactivation in these patients.

## Data Availability

All relevant data has been described in the case report.
